# From Natural Killer Cell Receptor Discovery to Characterization of Natural Killer Cell Defects in Primary Immunodeficiencies

**DOI:** 10.3389/fimmu.2019.01757

**Published:** 2019-07-24

**Authors:** Giovanna Tabellini, Ornella Patrizi, Kerry Dobbs, Vassilios Lougaris, Manuela Baronio, Daniela Coltrini, Alessandro Plebani, Raffaele Badolato, Luigi D. Notarangelo, Silvia Parolini

**Affiliations:** ^1^Department of Molecular and Translational Medicine, University of Brescia, Brescia, Italy; ^2^Laboratory of Host Defenses, Division of Intramural Research, National Institute of Allergy and Infectious Diseases, National Institutes of Health, Bethesda, MD, United States; ^3^Department of Experimental and Clinical Sciences, University of Brescia, Brescia, Italy

**Keywords:** natural killer cells, primary immunodeficiencies, monoclonal antibodies, functional assays, signaling transduction

## Abstract

Alessandro Moretta was Professor of Histology at University of Brescia from 1994 to 1997. It was in that period that we met and started a collaboration that continued in the years to follow. He immediately involved us in the production of monoclonal antibodies (mAbs) that allowed the identification and fine characterization of novel receptor molecules that were able to activate or inhibit human Natural Killer cell function, including several antibodies specific for Natural Cytotoxicity Receptor (NCR) and Killer-cell Immunoglobulin-like Receptor (KIR) molecules. These reagents, generated in our laboratory in Brescia, contributed to complete the studies aimed to characterize innate lymphoid NK cells, that had been initiated by Alessandro and his brother Lorenzo in Genoa. Soon, we identified an anti-KIR3DL2 that was subsequently shown to be helpful for the diagnosis and treatment of various forms of cutaneous T cell lymphoma. While in Brescia, Alessandro established a partnership with those of us who were working in the Department of Pediatrics; together, in short time we tackled the goal of studying the role of NK cells in patients with primary immunodeficiencies. This collaboration led to novel discoveries that shed light on the critical role played by NK cells in the immune response against virus and tumors in humans, as best exemplified by our characterization of the molecular mechanisms of impaired control of Epstein-Barr Virus (EBV) infection in patients with X-linked lymphoproliferative (XLP) disease. After Alessandro left Brescia to return to Genoa, our collaboration continued with the same enthusiasm, and even from a distance he remained an extraordinary example of an inspirational and generous mentor. This review is a sign of our gratitude to a mentor and a friend whom we deeply miss.

## Introduction

We (Silvia and Luigi) met Alessandro Moretta for the first time in 1994, when he became full Professor of Histology at the University of Brescia. He remained there until 1997, but we continued to collaborate with him even after his return to the University of Genoa. Since then, Alessandro was my mentor, inspiring my work and transmitting to me his passion for research. He immediately attracted my curiosity by sharing with me his passion on natural killer (NK) cells. In particular, at the time of his arrival in Brescia, Alessandro was working at the production of monoclonal antibodies (mAbs) directed against NK cells. He involved me in this project; together, we immunized mice with human NK cells that had been expanded *in vitro* with interleukin-2 (IL-2). Upon generating hybridomas from the spleen of treated mice, screening their supernatant, and isolating monoclonal antibody-producing cells through limiting dilution, we produced a large number of monoclonal antibodies directed against various NK cell receptor molecules. This work eventually resulted in the identification of novel surface molecules that modulate NK cell function. Furthermore, from the very beginning of this project Alessandro engaged another one of us (Luigi, also known as Gigi), with the aim of studying the role that NK cells may play in causing a higher risk of infections and malignancies in patients with primary immune deficiency (PID). In those years, the role of NK cells in human immune defense was not fully appreciated; in particular, it was known to what extent NK cell dysfunction contributes to the unique susceptibility to severe Epstein-Barr virus (EBV) infection that characterizes several forms of PID. If successful, these studies would help better understand human NK cells development, function and homeostasis, and could also shed some light on the development and function of other, less well-understood subsets such as adaptive and memory-like NK cells.

Over the year, the collaboration with Alessandro has been a wonderful journey that allowed us to discover the most intriguing aspects of NK cell biology.

## Generation of Different Isotypes of mAbs Specific for Receptor Molecules That Control and Regulate NK Cell Function

NK cells were originally identified on the capability of killing certain tumor cell lines in the absence of deliberate previous stimulation. More recently, it has become evident that NK cells play other important roles in immune responses, beyond cell-mediated cytotoxicity (1–3).

Upon engagement of various NK receptors and in response to certain cytokines, NK cells display regulatory functions that are especially important in the early inflammatory response that follows acute infection. After recruitment into peripheral tissues in response to chemokine gradients, NK cells must undergo a priming process in order to acquire full functional competence before migrating toward lymph nodes. NK cell priming takes place when they interact with other innate immunity cell types, that are either resident or that are recruited in peripheral tissues during inflammation, and that release a set of relevant cytokines. In addition, NK cell activity is enhanced by the recognition of virus-infected or tumor target cells (4).

A dynamic balance between inhibitory and activating NK cell receptors controls NK cell effector functions. NK cell activation can be restrained by various inhibitory receptors that include a family of strictly homologous surface molecules referred to as Killer-cell Immunoglobulin-like Receptor (KIRs) molecules, that recognize unique patterns of HLA (Human Leucocyte Antigen) class I alleles or, in the case of NKG2A/CD94 heterodimer, non-classical HLA-E alleles.

The nature and the number of ligands expressed by target cells for NK activating and inhibitory receptors is the main factor that determines susceptibility of such target cells to NK-mediated lysis (1–3). In cells undergoing viral infection or tumor transformation, alterations (and/or down-modulation) of HLA class I molecules that include either the whole HLA class I phenotype, or selected alleles, are frequently observed (5).

Since inactivation of NK cell function represents a central safety mechanism to prevent killing of self HLA class-I^+^ cells, it was necessary to postulate that in order to kill self HLA class-I^+^ cells under appropriate conditions (viral infection or tumor transformation), NK cells must express also activating receptors. In those years, Alessandro successfully identified three important activating NK receptors named Natural Cytotoxicity Receptors (NCRs) recognizing non-HLA ligands (6–11).

In addition to NCRs (NKp30, NKp44, and NKp46), NK cell activation can also be induced upon signaling through synergism of activating and costimulatory NK cell receptors including NKG2D, DNAX accessory molecule-1 (DNAM-1), 2B4, NTB-A, CD59, NKp80, CD2, and CD94/NKG2C. In fact, particular combination of activating receptor may trigger NK cell activation more efficiently than others. Moreover, NK cell activating receptors may differently recognize ligands expressed on target cells in qualitatively distinct events (12).

Alessandro Moretta described the NK cell subsets by his numerous publications in this way: “in human peripheral blood can be distinguished two NK cell populations characterized by different density of CD56 and CD16 expression on the cell surface: CD56^bright^CD16^−/low^ and CD56^dim^CD16^bright^ cells. These two NK cell subsets differ for the expression pattern of various other cell surface and chemokine receptors. A characteristic of immature, CD56^bright^ NK cells is the expression of high levels of NKp46, CD94/NKG2A, and CCR7. By contrast, mature CD56^dim^ cells express CXCR1 and KIRs at higher density. Furthermore, CD56^bright^ and CD56^dim^ NK cells have distinct functional properties, with CD56^bright^ cells being potent producers of cytokines, and CD56^dim^ cells being active mediators of natural and antibody-dependent cellular cytotoxicity, as also reflected by higher intracellular levels of perforin and granzymes. In healthy donors, CD56^bright^ cells comprise a minority (5–10%) of all circulating NK cells, but because they express CCR7, they can migrate to secondary lymphoid organs where they represent the predominant NK cell subset. A subset of CD56^dim^ KIR^+^ NK cells, expressing CD57 represent terminally differentiated NK cells, whereas a further subset expressing the CD56^−^ CD16^+^ CD57^+^ KIR^+^ phenotype were thought to represent exhausted NK cells and were characterized in HIV^+^ patients” (13–17).

When Alessandro arrived in Brescia, in collaboration with his brother Lorenzo, he had already produced several mAbs that had permitted to identify novel inhibitory and activating receptors and co-receptors of human NK cells. A first category of NK receptors identified with inhibitory and activating functions included p58 (KIR2DL1/L2) and p50 (KIR2DS1/S2) molecules, identified by EB6 and GL183 mAbs, respectively. These p58 and p50 molecules are part of the family of Killer Immunoglobulin-like receptors (KIRs), and they both recognize HLA-C.

**Figure 1:**
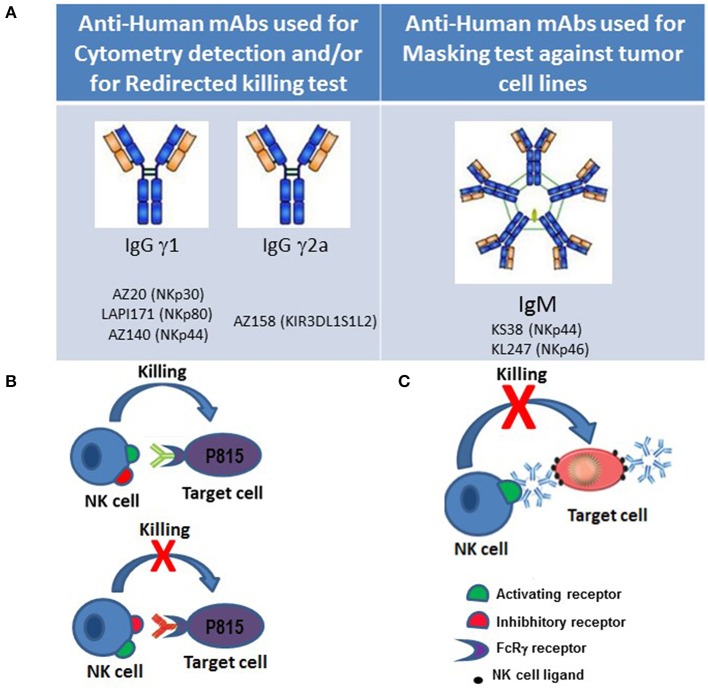
Approaches to functional studies of NK cell receptors. **(A)** Isotypes of monoclonal antibodies against NK cell surface molecules that were generated in our laboratory and used for flow cytometry and/or cytotoxic assays against tumor cell lines. **(B)** In redirected-killing assays, the murine mastocytoma FcγR^+^ P815 cell line is used as target. Binding of murine mAbs of γ1, γ2a, and γ2b IgG isotypes to NK receptors on one hand, and to the FcγR on P815 cells on the other, allows cross-linking of activating and inhibitory NK cell receptors, thereby permitting to study their role in NK cell-mediated cytotoxicity. **(C)** Anti-NK cell activating receptors of IgM isotype can mask receptor-ligand interactions blocking cytolysis of target cells.

In order to continue the studies on KIR molecules, after identifying molecules characterized by three domains KIR3DL1/S1/L2 (p70 and p140), Alessandro and I (Silvia), in Brescia, produced a novel mAb, named AZ158, that was able to recognize both p70 and p140 molecules. Important features of this were: first, that it binds the same epitope shared by the KIR3DL2 in either dimeric or monomeric form, as well as the monomeric KIR3DL1 and KIR3DS1, present as monomeric (p70) or dimeric (p170) form; second, that mAb AZ158 is of IgG2a isotype, and therefore different from the other anti-KIR3D mAbs that are of IgG1 isotype. This permitted to analyze NK cell subsets characterized by clonal expression of various KIRs through multi-fluorescence flow cytometry (Figure 1A).

Production of a reagent that specifically binds KIR3DL2 had immediate clinical translational implications. In fact, KIR3DL2 is expressed not only by NK cells, but in patients with mycosis fungoides and Sézary syndrome it is also expressed by malignant CD4^+^ T cells. For this reason Alessandro, in collaboration with M. Bagot and A. Bensussan and with Innate Pharma, developed clinical studies for the validation of KIR3DL2 as a tumor antigen in various forms of cutaneous T cell Lymphoma that led to a successful clinical trial (18) (2003 patent no. US 9, 828, 427 B2).

At a later time, three other non-HLA-specific receptors (NKp46, NKp30, and NKp44) were also discovered by Alessandro. These NCRs play a crucial role in the NK-mediated recognition and killing of most target tumor and/or virus-infected cell lines expressing NCR-ligands. NKp46 and NKp30 are expressed by both resting and activated NK cells, while the expression of NKp44 is restricted to activated NK cells (6, 9, 10). It is of note that NCRs display a strictly coordinated pattern of surface expression. In particular, they are collectively expressed by NK cells either at high or at low surface density; consequently, NK cells display either an NCR^bright^ or an NCR^dull^ phenotype (19). In the laboratory at the University of Brescia, we detected other mAbs specific for NCR molecules that helped perform biochemical studies to further characterize NCR function. In particular, production of mAb of IgM isotype to NKp44 and NKp46 (KS38 and KL247 mAbs, respectively) allowed us to perform NCRs masking experiments in the search of possible tumor ligands (Figure 1C). Specifically, the use of these IgM isotype antibodies allowed to identify the presence of the major or minor expression of NCR-ligands recognized by NK cells that showed different cytotoxic capability against various histological tumor cell lines. These masking experiments allowed to obtain new results on the functionality of NK cells and their receptors in addition to those already obtained with the redirected killing assays. In fact, in these tests, the cross-linking between NK receptor specific antibodies and FcγR expressed on P815 murine tumor cell, mimics receptor function while masking tests block receptor-ligand interaction (Figure 1B).

Moreover, we also generated mAbs against CD244, NTB-A, NKp80, DNAM-1, and CD59 co-receptor molecules. Use of these reagents in appropriate *in vitro* assays was critical to characterize the function of each of these molecules (20–23). The results obtained clearly demonstrated that differences in response do not reflect a functional heterogeneity of co-receptor expression but rather depend on the co-engagement of triggering receptors.

Finally, while Alessandro's brilliant imagination allowed us to produce such precious reagents and to obtain novel mechanistic insights into regulation of NK cell function, his generosity in sharing these products with the scientific community at large paved the way for a fine characterization of NK cell dysfunction in various forms of Primary Immunodeficiencies (PID).

## NK Cells and Primary Immunodeficiencies

NK cells are innate immune cytotoxic effector cells well-known for their role in antiviral immunity and tumor immunosurveillance. To date, a growing body of evidence indicates that NK cells play an important role in anti-viral immune responses. In particular, the study of patients suffering either from rare isolated NK cell deficiencies or more profound immunodeficiency syndromes has offered novel insights into NK cell biology. In this regard, PIDs provide unique opportunities for better understanding the specific role played by individual molecules in NK cell function and anti-viral immune response in particular, as also exemplified by the results of our exciting collaboration with Alessandro at the University of Brescia.

### Functional Analysis of Activating NK Cell Receptors in X-Linked Lymphoproliferative Disease (XLP1)

X-linked lymphoproliferative disease type 1 (XLP1) is a severe immunodeficiency that affects approximately 1 in 1 × 10^6^ males and is characterized by extreme susceptibility to EBV infection, leading to fatal infectious mononucleosis, lymphoma, aplastic anemia, and/or dysgammaglobulinemia, and a high rate of death early in life (24). In 1998, several groups discovered the genetic basis of XLP1 (25–27). In particular, the gene mutated in XLP1 is named Src homology 2 domain containing protein 1 A (*SH2D1A*), and encodes for a small adaptor molecule that plays a crucial role in signaling via a number of surface molecules expressed by various cells of the immune system (28). This intracytoplasmic polypeptide of 128 amino acids, also named SAP (Signaling lymphocyte-activating molecule-Associated Protein), is expressed by T lymphocytes and NK cells, but not by normal B cells. While it was logical to assume that abnormal function of cytotoxic T lymphocytes (CTLs) and possibly NK cells could play a role in the pathophysiology of XLP1 (29), the basis of selective susceptibility to EBV infection, and the role played specifically by SAP mutations in this process, remained unclear. In the attempt to address these questions, we studied NK cells from two XLP1 patients.

The surface molecules that bind to SAP include a group of receptors belonging to the Immunoglobulin Superfamily, and in particular two NK cell co-receptors with activating function: 2B4 (CD244) and NTB-A, a novel surface molecule that had been recently identified by Alessandro's group (22, 28, 30).

**Figure 2:**
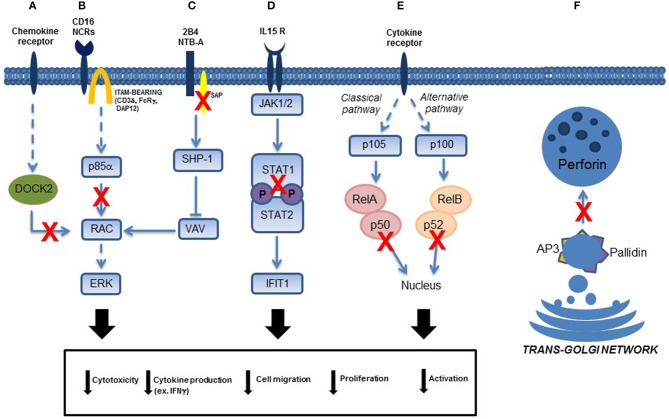
Schematic representation of NK cell transduction pathways involved in primary immunodeficiencies. Functional NK cell defects due to: **(A)** *DOCK2* mutations, **(B)** p85α (*PIK3R1*) mutations, **(C)** SAP (*SH2D1A* gene) mutations in patients affected by XLP1, **(D)** STAT1 mutations, **(E)** p50 (in the canonical pathway of NF-κB signaling) and p52 (in the alternative pathway of NFκB signaling) mutations, and **(F)** AP-3 and Pallidin mutations in patients affected by Hermansky-Pudlak type 2 and 9, respectively. Direct and indirect effects on signaling are represented with continued or dotted lines, respectively.

Human 2B4 is a 70 kD glycoprotein expressed on NK cells and its engagement by mAb-mediated cross-linking of 2B4 or by its ligand CD48 resulted in enhancement of cytolytic activity. Because 2B4 is a co-receptor, its ability to induce NK cell activation is dependent upon the co-engagement of main triggering NK cell receptors including NKp46 (20). So, upon mAb-mediated engagement, 2B4 undergoes tyrosine phosphorylation and associates SAP. Under normal conditions, this association appears to be crucial for SHIP-1 displacement and for the transduction of the activating signals (Figure 2C) (28, 30).

Various studies had suggested that “SAP could act as a regulator of signaling by competitive displacing of SHP molecules from the cytoplasmic tails of the receptor. Thus, SAP was likely to prevent the generation of SHP-mediated inhibitory signals (28, 30). In our work, we reported that 2B4 function was dramatically altered in XLP1 patients carrying non-sense *SH2D1A* mutations. We demonstrated that in such patients 2B4 associates with SHIP-1, and transduces inhibitory signals. Importantly, CD48, the ligand of 2B4, is up-regulated in EBV–infected B cells (28, 30). Thus, engagement of 2B4 by CD48 determined not only an inhibition of NK-mediated lysis, but also a shutdown of activating signals engaged by other ligands in addition to CD48 recognized by NK cells, leading to the complete inability to kill EBV^+^ targets” (30). Remarkably, the cytolytic activity of XLP1-NK cells against EBV-infected targets could be restored by mAb-mediated disruption of the 2B4/CD48 interactions. Altogether, the altered function of 2B4 may account for a general inability of different cytolytic effector cells to control EBV-infection.

Soon thereafter, Alessandro's research group discovered a novel 60-kD glycoprotein, termed NTB-A that is expressed by human NK, T, and B lymphocytes. Similar to 2B4 (CD244), NTB-A belongs to the CD2 subfamily of immunoglobulin-like molecules, and functions as an activating co-receptor. Its structure is characterized by a cytoplasmic domain containing three tyrosine-based motifs that, when NTB-A undergoes tyrosine phosphorylation, allow binding to SAP. Interestingly, in XLP1 patients, NTB-A, similar to 2B4, transduces inhibitory rather than activating signals. Furthermore, mAb-mediated masking experiments suggested that NTB-A may recognize cell surface ligand expressed on EBV-infected B lymphocytes. To further illustrate the critical role played by 2B4 and NTB-A mediated inhibitory signals in the pathophysiology of XLP1, mAb-mediated masking of these molecules resulted in virtually complete restoration of XLP1-NK cell-mediated cytotoxicity against EBV-infected target cells (22, 28, 30).

### Hermansky Pudlak Syndromes Associated to AP-3 Complex and Pallidin Mutations

Pigmentary dilution disorders recognized as immunodeficiencies are characterized by partial albinism of hair, skin, and eyes, together with leukocyte defects. These disorders include Chediak-Higashi, Griscelli, Hermansky-Pudlak (HPS), and MAPBP-interacting protein deficiency syndromes (31, 32). These are recessive autosomal gene defects in which proteins with specific roles in the biogenesis and trafficking of secretory lysosomes are encoded. These intracellular granules are essential constituents of melanocytes, platelets, granulocytes, CTLs, and NK cells. Among nine genetically distinct type of HPS, only type 2 (HPS2) and type 9 (HPS9) are characterized by immunodeficiency (32).

In 2000, we analyzed two siblings who presented with oculocutaneous albinism, neutropenia, recurrent infections, and bleeding disorder. Initially, we noticed a severe defect of their NK cell cytotoxicity, but it was only some years later that we discovered that they carried a mutation in the *AP3B1* gene, encoding the β3A subunit of the heterotetrameric adapter protein (AP-)3 complex. Defects of the β3A subunit compromise stability of the entire AP-3 complex involved in cellular protein trafficking (Figure 2F). The absence of the AP-3 complex impairs regulated movement of protein sorting to secretory lysosomes. Consequently, regulation and function of cells related to secretory lysosomes are defective. Besides functional defects of melanocytes, platelets and CTLs, we observed impaired differentiation of neutrophils, NKT, and NK cells. In particular NK cells had reduced lysosomal pools of perforin, but normal levels of granzyme (33, 34).

After some years, the diagnosis of nodular lymphocytes predominance type Hodgkin lymphoma (NLPHL) was made in the same two young siblings affected with HPS2. By analyzing their peripheral blood immune cells, we found that CTL, NK, and iNKT cells from these patients were significantly impaired in their number and function, including tumor cell killing activity (35). The development of NLPHL suggested a possible involvement of effector function of multiple cell types including NK cells. In particular, we noted a significant reduction in the proportion of CD56^bright^ NK cells, and high expression of the NK cell maturation marker CD57 on CD56^dim^ cells, in spite of reduced intracellular perforin content. We linked the high expression of CD57^+^CD56^dim^ NK cells to an expansion of a human “memory NK cells” subset, reflecting the history of recurrent viral infection in these patients, as also demonstrated previously by various authors (36). Even more important to explain the complications of their HPS2, was the demonstration of a severe NK cell cytolytic defect combined with a reduced production of IFN-γ after engagement of NK cell activating receptors. In fact, NK cells from the HPS2 patients failed to properly recognize and kill a large series of tumor cell lines *in vitro*, including Hodgkin Lymphoma cell lines (35).

Thereafter, we analyzed one HPS9 patient who manifested partial albinism, nystagmus, normal neurological development, and absence of platelet delta granules but, at variance with what observed in HPS2, the patient lacked coagulation defects (37). HPS9 is the most recently defined subtype of HPS and is caused by biallelic mutations of the *PLDN* gene, encoding for pallidin, a component of the biogenesis of lysosome-related organelles complex-1 (BLOC1). In the patient with HPS9, *PLDN* mutations led to undetectable expression of the protein product in NK cells. Comparative analysis of NK cells from the patient and healthy controls, showed that pallidin regulates the expression of lysosomal membrane proteins. In particular, freshly isolated NK cells from the patient expressed higher levels of CD107a (a marker of NK cell degranulation) and of CD63 on the cell surface; after *in vitro* activation, NK cell degranulation (measured by change in CD107a surface expression) against NK-susceptible erythroleukemia K562 and B-EBV 221 cell lines was reduced in the patient's NK cells as compared to what observed in controls, indicating impaired trafficking of cytolytic granules to the plasma membrane (37). These defects were similar, although not as severe, to those observed in HPS2.

### Defective Activity of NK Cells Due to Abnormalities in Intracellular Signaling in Patients With *STAT1, NFKB1, NFKB2*, and *PIK3R1* (PI3K p85α) Gene Defects

In more recent years, Alessandro strongly encouraged us to investigate the role of NK cells in other forms of PID, and in particular in those with increased susceptibility to viral infections. His continuous support was critical for the discovery of previously unrecognized NK cell defects in a variety of PIDs.

#### STAT1 Mutations

Subjects affected by Signal Transducer and Activator of Transcription 1 (STAT1) deficiency suffer from life-threatening bacterial, mycobacterial, viral, and fungal infections. Complete STAT1 deficiency is inherited as an autosomal recessive disease; partial STAT1 deficiency is inherited as an autosomal recessive or autosomal dominant trait. We described a patient with homozygous STAT1 splicing mutation leading to skipping of exon 3 who developed generalized mycobacterial infections and severe viral disease sustained by cytomegalovirus (CMV) (38). We reported that the patient's cells displayed a complete defect of STAT1 DNA-binding activity after stimulation with IFN-γ and IFN-α, and failed to respond even to high doses of these cytokines. These biologic defects were associated with a partial impairment of NK cell function, which could contribute to the increased susceptibility to infections with viral and intracellular pathogens. We observed that unstimulated NK cells displayed a cytotoxicity defect against K562 target cells. It is probable that the defect of NK-mediated cytolysis was related to the immunomodulatory role of STAT1 because NK cytotoxicity is regulated by cytokines that require STAT1 for signaling. Moreover, we observed that IFN-γ production by STAT1-deficient NK cells activated with IL-15 and IL-12 in the presence of target cells, was impaired, suggesting that the IL-15 response involves STAT1 signaling (38) (Figure 2D).

Based on these observations, we studied NK cells in 8 patients with *STAT1* gain-of-function (GOF) mutations. This condition was initially reported to cause chronic mucocutaneous candidiasis, but was subsequently shown to cause also recurrent bacterial and viral infections. We reported that “patients with *STAT1* GOF mutations display abnormal NK cell function and proliferation (39). Upon *in vitro* activation with IL-2, NK cells from these patients show impaired cytolytic activity. Moreover, the defect of NK cytotoxic activity observed in patients with *STAT1* GOF mutations was not related to abnormal expression/function of NK receptors or to reduced perforin expression, but rather to an impaired response of these cells to IL-2 or IL-15. Indeed, NK cells from the patients produced lower than normal levels of IFN-γ after stimulation with IL-15, but normal levels upon stimulation with IL-12 and IL-18 (39). This suggests that the NK cytotoxicity defect detected also in these patients was probably related to abnormal response to the immunomodulatory cytokines IL-15 and IL-2.” Interestingly, Tabellini et al. showed that “NK cells from patients with *STAT1* GOF mutations, manifested enhanced STAT1 phosphorylation in response to either IL-15 or IL-2 stimulation, whereas a weak signaling is detected in normal NK cells in response to IL-2 or IL-15, even after prolonged stimulation. This suggests that *STAT1* GOF mutations can interfere with important steps in the differentiation and functions of T and NK cells, resulting in impaired generation of TH17 cells and reduced proliferation of NK cells” (39). In addition, the observation that *in vitro* activated NK cells from patients with *STAT1* GOF mutations produce reduced amounts of IFN-γ, suggest that this defect may also lead to increased susceptibility to intracellular pathogens. Overall, our data indicate that abnormalities of NK cell function may play an important role in determining the clinical phenotype of this condition (39).

#### NFKB1 and NFKB2 Gene Defects

The NF-κB (NF-kappaB: nuclear factor of kappa light polypeptide gene enhancer in B cells) signaling pathways play an important role both in the innate and in the adaptive immune system (40).

The NF-κB transcription factor family consists of five members: NF-κB1, NF-κB2, RelA, RelB, and c-Rel. We had the chance to study patients with mutations of the *NFKB1* gene (that encodes the precursor p105 which is processed to the mature p50) and one patient with *NFKB2* mutation, whose gene encodes the precursor p100 and the mature p52.

Two pathways of NF-κB signaling have been described. The canonical pathway, which includes NF-κB1, mediates numerous immunological and inflammatory cellular responses, whereas the non-canonical pathway, which involves NF-κB2, has more restricted immunological functions mainly focusing on B cell homeostasis and regulation of self-tolerance by medullary thymic epithelial cells (Figure 2E).

The NF-κB1 and NF-κB2 proteins were recently reported to be mutated in a limited number of common variable immunodeficiency (CVID) patients, some of which were analyzed by our group (41, 42). CVID is the most common symptomatic primary immunodeficiency characterized by low immunoglobulin serum levels, low vaccine responses, and recurrent infections. Several genetic mechanisms have been reported to account for CVID in the last few years, and involve mutations in CD19, MS4A1 (CD20), CR2 (CD21), ICOS, TNFRSF13C, TNFRSF13B, PLCG2 (phospholipase Cg2), CD81, LRBA, and PRKCD (protein kinase CD) as well as in NF-κB1 and NF-κB2.

We had the opportunity to study one patient affected with CVID due to a *de novo* heterozygous non-sense mutation (p.Arg853^*^) in *NFKB2* and demonstrated impaired NK cell cytotoxic activity despite normal NK cell counts and normal expression of different NK receptors (41). Moreover, we observed a normal expression of the maturation marker CD57 on CD56^dim^ NK cells. This is the first description of impaired NK-cell activity associated with *NFKB2* mutations. These findings broaden the immunologic defects in NF-κB2 deficiency, confirm the heterogeneous and complex immunologic and clinical phenotype in disorders in which NF-κB components are defective, and underline an important role for NF-κB in NK-cell cytotoxic activity.

Subsequently, we had the possibility to analyze NK cells from patients with NF-κB1 deficiency (42). This was especially important, since until then, data on the role of NF-κB signaling in NK cells were largely limited to observations in mice. In particular, we provided evidence that monoallelic *NFKB1* mutations affect both maturation and effector functions of human NK cells. In fact, *NFKB1*-mutated NK cells showed reduced percentage of CD56^+^ NK cells expressing KIRs, NKp46, CXCR1, CCR7, and CD16, when compared to healthy controls. Expression of CD57, a classical maturation marker, was also downregulated. By studying CD56^bright^ and CD56^dim^ NK cells, we noticed a similar proportion of CD56^dim^ CD57^low^ cells in patients and controls. In contrast, the population of more mature CD56^dim^ CD57^bright^ NK cells was significantly reduced in individuals with *NFKB1* mutations (42). These results, described in our work, indicate an impaired peripheral maturation of NK cells in patients with *NFKB1* haploinsufficiency and they were supported by a significant reduction of CD62L (an additional marker of maturation) on the CD56^dim^ CD57^+^ NK cell subsets, as well as accumulation of CD56^bright^ CD62L^+^ NK cells, a finding not observed in healthy controls. Together, these data suggest that the canonical NF-κB pathway orchestrates unique aspects of human NK cell maturation stages (42).

Finally, *NFKB1*-mutated NK cells showed impaired cytotoxicity, IFN-γ production, and proliferation. These observations indicate that defective maturation and function of NK cells may play an important role in the increased susceptibility to viral infections that has been reported in NFκB1 haploinsufficient patients (42).

#### PIK3R1 (p85α) Defects

Recently, novel insights indicating the role that PI3K signaling plays in human NK cell maturation and lytic function are suggested by the identification of patients with phosphoinositide-3-kinase (PI3K)-signaling pathway mutations that can cause primary immunodeficiency (43). Class I PI3Ks are divided into class IA (p110α, p110β, p110δ) and class IB (p110γ) kinases, which interact with the regulatory subunits p85α, p50α, p55α, p85β, and p55γ (for class IA kinases), and with p101 and p84 (for class IB kinases) (44). Many authors showed an involvement of PI3K in multiple functions of NK cell biology, including development/maturation, homing, priming, and function. In human NK cells, the PI3K-signaling pathway plays a direct role in signaling downstream from activating receptors, including 2B4 and KIR receptors (45–47). The recruitment of p85α, in combination with Grb2, is also necessary and sufficient for the propagation of signaling, promoting cytotoxicity upon engagement of NKG2D associated with the DAP10 adaptor (47). In addition, “PI3K activates a Rac1–MEK–ERK pathway that is a key signaling pathway for actin reorganization and cellular polarization (48) (Figure 2B). The central role of PI3K in mediating cell polarization is consistent with PI3K-induced activation of CDC42 at the NK cell immune synapse; in particular, p85α acts as a scaffold to target and position PI3K, and subsequent recruitment of guanine nucleotide exchange factors to the membrane (49). As such, the role of PI3K signaling in cytotoxicity and NK cell migration can be through the control of actin remodeling, polarization, and even granule exocytosis, which requires intracellular calcium store mobilization” (50).

Following the discovery of *PIK3CD* GOF mutations, that result in constitutive p110δ activation in patients with Activated PI3K Delta Syndrome type 1 (APDS1) (51), monoallelic GOF *PIK3R1* mutations were identified in 12 patients affected with a hyper–IgM-like primary immunodeficiency/immune dysregulation condition associated with T- and B-cell maturational and functional defects (52). These mutations deprive p85α from its regulatory function, unleashing p110δ, and thereby causing APDS type 2 (ADS2) (51). However, when the first cases of APDS2 were described, the impact of this p85α mutant protein on the maturation and function of human NK cells had not yet been studied. To address this issue, we investigated NK cell phenotype and function in two patients with APDS2 (53).

We showed that NK-cell maturation from *PIK3R1*-mutated patients is normal (53). This is in contrast with maturational defects described in the animal model. We then tested NK cell function, including degranulation, IFN-γ secretion, and cytolytic activity against of EBV-infected target cells (53).

Upon IL-2 stimulation, patients' NK-cell degranulation against the human erythroleukemia cell line K562 was significantly reduced as compared to what observed with healthy control NK cells. This impairment was also confirmed by a classical chromium release killing assay, underscoring an important role for p85α in this process in humans, similar to what was observed in p85α knockout mice (53). Moreover, IFN-γ production was significantly reduced in patients' as to healthy control NK cells. After demonstrating that NK cell activating receptors function normally in APDS2 patients (as shown by redirected killing assays), we observed that IL-2 activated NK cells from these patients display reduced degranulation against the 721.221 EBV^+^ lymphoblastoid cell line, and failed to upregulate CD107a upon engagement of autologous EBV-infected B cells (53). These results indicate an essential role for p85α in human NK-cell lysis of EBV-infected cells, thereby providing mechanistic insights into the increased susceptibility to EBV infection and EBV-associated lymphoproliferative disorders that patients with APDS1 and APDS2 manifest (53).

### Impaired NK Cell Function in Patients With DOCK2 Deficiency

A few years ago, we performed genetic and immunological investigations in five unrelated children of different ethnic origin who manifested severe susceptibility to infections sustained by a broad spectrum of pathogens. The immunological phenotype of the patients included severe lymphopenia and functional defects affecting T, B, and NK cells. Through whole exome sequencing, we established that these patients carried bi-allelic mutations in the *DOCK2* gene (54).

DOCK2 is a large protein involved in intracellular signaling network, that specifically activates isoforms of the small G protein RAC in response to engagement of various cell surface receptors, including T and B cell receptors, chemokine receptors, and various NK cell receptors (Figure 2A). We demonstrated that T, B, and NK cells from the patients manifest defective chemokine-induced migration and actin polymerization, and that RAC1 activation was impaired in T cells (54).

We also showed that DOCK2 deficiency impairs NK-cell degranulation against K562 target cells. The ability of NK cells to fight viral infections and the onset of tumors correlates with the functionality of a variety of activating NK cell receptors interacting with the distinct adaptor (DAP10, DAP12) and signaling (CD3ζ and FcεRγ) molecules (55). Furthermore, triggering of activating NK cell receptors induces actin polymerization, phosphatidylinositol-3-OH kinase activation and phosphorylation of MEK and ERK, ultimately promoting NK-cell cytotoxicity. Based on this knowledge, we analyzed NK cells from DOCK2 deficient patients. In particular, we studied NK cell degranulation upon engagement of CD16, NKp30, NKp46 (all of which utilize CD3ζ and FcεRIγ for signaling), or NKG2D (which recruits the DAP10 adaptor), and observed severely impaired degranulation in a patient, and moderately impaired in another patient with residual amounts of DOCK2 protein. Degranulation was also impaired in patient-derived IL-2 activated polyclonal NK cells upon engagement of NKp44 (which utilizes DAP12) (54). We observed reduced levels of F-actin in patient NK cells upon CD16 and NKp46 stimulation, reminiscent of similar observations in *Dock2*^−/−^ mice reflecting impaired tonal signaling through antigen and chemokine receptors. Moreover, we noticed reduced phosphorylation of ERK1/2 and MEK, and impaired actin polymerization in polyclonal NK cells derived from patients upon cross-linking of activating receptors. Finally, we showed that upon stimulation with IL-12 and IL-18, the proportion of NK cells expressing IFN-γ was markedly reduced in patients when compared with IFN-γ production in normal NK cells (54). These data demonstrated that DOCK2 deficiency is a combined immunodeficiency affecting the function of multiple white blood cell types, including NK cells, further supporting the notion that hematopoietic stem cell transplantation (HSCT) should be considered soon after diagnosis to prevent life-threatening infections and early death.

### Altered Phenotype and Function of NK Cells Derived From Patients With RAG and NHEJ Defects

In the last years of collaboration with us, Alessandro had shown much interest in a research proposed by Gigi concerning the phenotypic analysis of NK cell subsets in patients carrying mutations in the recombinase-activating genes *RAG1* and *RAG2*. The RAG1 and RAG2 proteins play a critical role in V(D)J recombination and therefore in T and B cell development, but are dispensable for the development of NK cells (56). Consistent with this, null mutations in the RAG genes are associated with T^−^ B^−^ NK^+^ severe combined immune deficiency (SCID). However, we and others have shown that hypomorphic mutations in humans are associated with a broad range of clinical and immunological phenotypes, whose severity correlates with the residual recombination activity of the mutant RAG proteins (57). Nonetheless, irrespective of the phenotype, human RAG deficiency is associated with a dismal prognosis, with death early in life in infants with SCID, and severe infections, autoimmunity and inflammation later in life in patients with leaky forms of the disease. Therefore, HSCT represents the mainstay of treatment for patients with RAG deficiency; however, as compared to other forms of SCID and related diseases, HSCT for RAG deficiency is associated with a high rate of graft rejection (58, 59). Based on the observation that NK cells play an important role in graft-vs.-leukemia and graft rejection after HSCT (40), it had been hypothesized that they may also contribute to the increased risk of graft rejection after unconditioned HSCT for RAG deficiency. However, why this would be true for RAG deficiency and not for other forms of NK^+^ SCID, remained unclear.

Although RAG genes are not required for NK cell development, data in mice indicated that RAG deficiency affects NK cell phenotype and function. It had been shown that expression of the *Rag* genes begins in common lymphoid progenitor cells that give rise to T, B, and NK cells (60). A seminal work by Joe Sun at Memorial Sloan Kettering had shown that NK cells from Rag-deficient mice have an abnormally activated phenotype and display enhanced cytotoxicity, associated with reduced cellular fitness (61). The hypothesis was put forward that *Rag* gene expression in common lymphoid progenitor cells provides preferential advantage to cells with superior DNA repair capacity, as suggested by mixed bone marrow chimera transplantation experiments, in which Rag-sufficient NK cells outcompeted Rag-deficient cells (56).

These observations paved the way to explore the hypothesis that NK cells from RAG-deficient patients could be dysfunctional, and that their possibly hyperactivated status might play a role in promoting rejection after HSCT. In collaboration with Alessandro and taking advantage of a large repository of specimens from patients with RAG deficiency and other forms of PID, we decided to test this hypothesis.

With his help and generosity, we utilized a large panel of mAbs (many of which he had generated) that define various steps in the differentiation process from CD56^bright^ to CD56^dim^ cells to study NK cells in 66 patients with defects in the *RAG* genes or in other genes involved in non-homologous end joining (NHEJ) genes (*DCLRE1C, LIG4, NHJ1*), or with other forms of PID with selective T or B cell deficiency unrelated to defects in VDJ recombination and DNA repair, and in healthy donors of comparable age (62). In our research, we observed that “NK cells from patients with mutations in *RAG* and NHEJ genes have an immature phenotype, with significant expansion of CD56^bright^ CD16^−/int^ NKG2A^+++^ CD57^−^ cells, and a reduced percentage of CD56^dim^ CD16^hi^ cells expressing CD57, KIRs, and CXCR1 than observed in age-matched healthy controls (62). These observations suggest that NK cells from patients with RAG/NHEJ defects have a more immature phenotype when compared to age-matched healthy controls and to SCID not due to RAG/NHEJ defects. These data contrast with findings in *Rag*^−/−^ mice whose peripheral NK cells display a more mature phenotype (62). However, we found that in spite of their immature phenotype, NK cells from patients with RAG/NHEJ defects have enhanced degranulation capacity and express higher amounts of perforin as compared to control NK cells.” This hyperactivation status of NK cells resembles what observed in *Rag*^−/−^ mice (56) and may indeed contribute to enhanced graft rejection activity after HSCT. Inclusion of serotherapy targeting NK cells in the HSCT conditioning regimen for RAG deficiency may therefore be beneficial to reduce the risk of graft rejection.

At the same time, because many of the patients included in our study suffered from CMV infection, this provided an opportunity to assess whether these patients have an increased proportion of “memory” NK cells (63), a topic that Alessandro was particularly interested in. It is well-known that CMV can drive expansion of NKG2C^+^ NK cells that produce high amounts of IFN-γ as compared to NKG2C^+^ NK cells from CMV-seronegative subjects (64). These NKG2C^+^ NK cells may represent “memory” NK cells capable of prompt responses upon secondary exposure to CMV. We failed to observe an expansion of NKG2C^+^ NK cells in CMV-infected patients with RAG/NHEJ defects. It is possible that T cells (that are defective in number in these patients) be required help in this process and for controlling CMV infection.

## Conclusions

The study of NK cell phenotype and function in patients with PID has provided unanticipated mechanistic insights into the pathophysiology of these diseases, and offered important novel information on the role of individual molecules in human NK cell biology. This long journey, which we have embraced with enthusiasm and dedication, would have not been possible without Alessandro's thoughtful guidance, scientific curiosity, and generosity. We remain inspired by his work, and will also miss his irony, the best remedy at times when our work seemed lost on a dead track.

## Author Contributions

SP and LN contributed conception and design of the study and wrote the first draft of the manuscript. GT, OP, KD, VL, MB, DC, AP, and RB wrote sections of the manuscript.

### Conflict of Interest Statement

The authors declare that the research was conducted in the absence of any commercial or financial relationships that could be construed as a potential conflict of interest.
